# Intravitreal conbercept for choroidal neovascularisation secondary to pathological myopia in a real‐world setting in China: Intravitreal conbercept was safe and effective in treating myopic choroidal neovascularization

**DOI:** 10.1186/s12886-021-01877-8

**Published:** 2021-03-04

**Authors:** Xin Nie, Yulong Wang, Hong Yi, Yanbin Qiao

**Affiliations:** Department of Ophthalmology, Chongqing General Hospital, University of Chinese Academy of Sciences, Pipashan 104, Yuzhong Qu, 400014 Chongqing, P.R. China

**Keywords:** Pathologic myopia, Choroidal neovascularization, Anti-VEGF therapy, Conbercept

## Abstract

**Background:**

To evaluate the 12-month efficacy and safety of intravitreal conbercept for myopic choroidal neovascularization (CNV).

**Methods:**

A retrospective, observational study. Thirty-four eyes of 34 pathologic myopic patients with CNV were treated with intravitreal conbercept (IVC) 0.5 mg with a follow up of 12 months. After the first injection, administration of conbercept followed a pro re nata (PRN) regimen. Outcomes included best corrected visual acuity (BCVA), central retinal thickness (CRT), CNV size, the total number of treatments, and adverse events.

**Results:**

The mean patient age was 55.88 ± 16.17 years, and the mean eye spherical equivalent was − 8.72 ± 3.75 D. The mean number of IVC over 12 months was 2.12 ± 0.69. Overall, best-corrected visual acuity(BCVA)improved from 0.86 ± 0.33 logMAR at baseline to 0.44 ± 0.32 logMAR at month 12 (*p* < 0.001), mean improvement of vision was 4.12 ± 2.69 lines. Mean central retinal thickness reduced from 285.9 ± 104.6 µm at baseline to 192.1 ± 97.5 µm at month 12 (*p* < 0.001). Mean CNV size decreased from 0.52 ± 0.38 mm^2^ at baseline to 0.31 ± 0.19 mm^2^ at 12 months (*p* < 0.05). All the 34 eyes had reduced or stable size of CNV. Thirty-two eyes (94.12 %) showed the absence of CNV leakage at the end of the study period. No severe systemic or ocular adverse events were observed.

**Conclusions:**

Intravitreal conbercept 0.5 mg was safe and effective for treatment of myopic CNV over 12 months in a real-world setting.

## Background

Myopic choroidal neovascularization (CNV) is a common cause of vision impairment in young and middle-aged patients with pathologic myopia (PM) in China [1, 2]. It has been reported that approximately 5 to 11 % of patients with PM will develop myopic CNV [1–3]. Previously, the standard treatment option for myopic CNV was photodynamic therapy (PDT). However, the patients treated with PDT have limited long-term visual outcomes [4]. Recently, anti-vascular endothelial growth factor (anti-VEGF) drugs such as ranibizumab, [5, 6] bevacizumab, [7] and aflibercept [8] have been used to treat myopic CNV with promising results. A study of PDT versus intravitreal bevacizumab (IVB) provided evidence for the superiority of IVB over PDT in treating myopic CNV [9]. The RADIANCE study demonstrated that intravitreal ranibizumab (IVR) provided significant visual improvement over PDT in patients with myopic CNV [5]. In the MYRROR trial, intravitreal aflibercept (IVA) was shown to be safe and effective in treating myopic CNV [8]. Anti-VEGF agents are now considered as first-line therapy for subfoveal and juxtafoveal myopic CNV [10, 11].

Similar to aflibercept, conbercept is an engineered protein that contains the extracellular domain-2 of the VEGF receptor 1 (VEGFR-1), and extracellular domains-3 and − 4 of the VEGFR-2. The structural difference between conbercept and aflibercept is that conbercept also contains the fourth binding domain of VEGFR-2, which is essential for receptor dimerization and enhances the association rate of VEGF to the receptor [12, 13]. Although only a few studies employing conbercept to treat myopic CNV have been reported, the effect of conbercept in patients with neovascular age-related macular degeneration (nAMD), polypoidal choroidal vasculopathy (PCV), macular edema after retinal vein occlusion (RVO) and diabetic macular edema (DME) are promising [14–17]. Conbercept was approved to treat myopic CNV by the State Food and Drug Administration of China in May 2017. However, there have been very few patients treated with intravitreal conbercept for myopic CNV. This retrospective study aimed to evaluate the 12-month outcomes of Chinese patients with myopic CNV treated with conbercept in a real-world setting.

## Methods

### Patients

This study was approved by the medical ethics committees of Chongqing General Hospital, and was performed in compliance with the 1964 Declaration of Helsinki. We retrospectively reviewed the medical records of 34 eyes from 34 patients with myopic CNV who were treated with intravitreal conbercept (IVC) with a follow-up of 12 months between August 2017 and March 2019.

At baseline and during all subsequent visits, every patient underwent a complete ophthalmic examination, which included best-corrected visual acuity (BCVA), tonometry, biomicroscopy, dilated fundus examination, and OCT (Cirrus; Zeiss, Germany). Fluorescein angiography (FA) was performed before the IVC, and also at 1, 2, 3, and 12 months after the IVC. Furthermore, FA was conducted to confirm the presence of fluorescein leakage in the macular area. The size of CNV was measured in the early-phase FA images using AutoCAD 2007 software in the fundus camera (F-10; NIDEK, Japan).

Patients were included in the study if they were aged ≥ 18 years and had high myopia (spherical equivalent ≤ − 6.0 diopters or ocular axial length ≥ 26.5 mm). In addition, all patients had to have an active subfoveal or juxtafoveal myopic CNV (as documented by spectral-domain OCT [SD-OCT] and FA) and a BCVA of 20/800 or better at baseline. Patients were excluded if they had any one of the following: (1) history of stroke; (2) CNV was secondary to causes other than pathologic myopia; (3) presence of any other ophthalmic diseases; (4) history of treatment with anti-VEGF drugs; (5) prior laser therapy or other intraocular surgery in the studied eye; or (6) intraocular pressure (IOP) ≥ 25 mmHg.

### Treatment procedure

Eligible patients received an intravitreal injection of conbercept of 0.5 mg at baseline. Patients then received more injections, as needed, following a pro-re-nata (PRN) schedule. Patients were examined each month for the first 3 months after the injection, and then every three months during the remainder of the follow-up period. Re-treatment was not performed unless any of the following was present in the studied eye: reduction in BCVA by at least 1 Snellen line; increase of at least 100 µm in central retinal thickness (CRT) on OCT; new, recurrent, or persistent subretinal or intraretinal fluid; CNV leakage on FA; or new macular hemorrhage.

### Study outcomes

The primary objective was to measure changes in BCVA from baseline to month 12 in patients with myopic CNV receiving IVC 0.5 mg. Secondary objectives included the following measures: changes in CRT on OCT from baseline to month 12; changes in CNV lesion size on FA from baseline to month 12; number of conbercept injections needed during the 12-month study period; ocular and systemic adverse events resulting from the injection at every study visit.

### Statistical analysis

The BCVA was assessed using the Snellen chart at a distance of 6 m and was converted to logarithm of the minimum angle of resolution (LogMAR) for analysis. A change of 0.1 LogMAR was considered a change of one line. Data were presented as mean values ± standard deviations (SD). Statistical significances of the differences from baseline to 12 months in BCVA, CNV size, and CRT were determined by Wilcoxon signed-rank test, Fisher^**’**^s exact test and paired *t*-test. Calculations were performed in SPSS Version 17.0 (SPSS, Chicago, IL, USA). A 푃 value of less than 0.05 was regarded as statistically significant.

## Results

### Baseline characteristics

Thirty-four eyes of 34 patients with active subfoveal or juxtafoveal myopic CNV were included and treated with at least 1 intravitreal injection of conbercept in this study. A 12-month follow-up was completed in all patients. The baseline and clinical characteristics of these patients are shown in Table 1. The mean age of the 34 patients was 55.88 ± 16.17 years with a range of 19 to 78 years. The average spherical equivalent was − 8.72 ± 3.75 D with a range of − 6.50 to − 18.00 D; the average axial length was 27.35 ± 1.23 mm with a range of 26.50–32.52 mm. The CNV was found in 18 right eyes (52.9 %) and in 16 left eyes (47.1 %). All patients with CNV were presented with a predominantly classic lesion in the macular area. The mean BCVA in LogMAR was 0.86 ± 0.33, the mean CRT at baseline was 285.9 ± 104.6 µm, and the mean size of the CNV before IVC was 0.52 ± 0.38 mm^2^.

**Table 1:** Baseline Patient Demographics and Clinical Characteristics

Characteristic	Patients, *N* = 34 (eyes, *N* = 34)
Mean age (SD), yrs	55.88 (16.17)
Sex, n (%)	
Male	22 (64.71)
Female	12 (35.29)
Eye side, n (%)	
Right	18 (52.94)
Left	16 (47.06)
Mean BCVA (SD), logMAR	0.86 (0.33)
Mean CRT (SD), µm	285.9 (104.6)
Mean CNV size (SD), mm^2^	0.52 (0.38)
Mean IOP (SD), mmHg	15.26 (3.21)
Mean axial length (SD), mm	27.35 (1.23)
Mean refraction-sphere (SD), diopters	− 8.72 (3.75)
CNV location, n (%)	
Subfoveal	30 (88.23)
Juxtafoveal	4 (11.76)

### Visual and anatomical outcomes

**Fig. 1:**
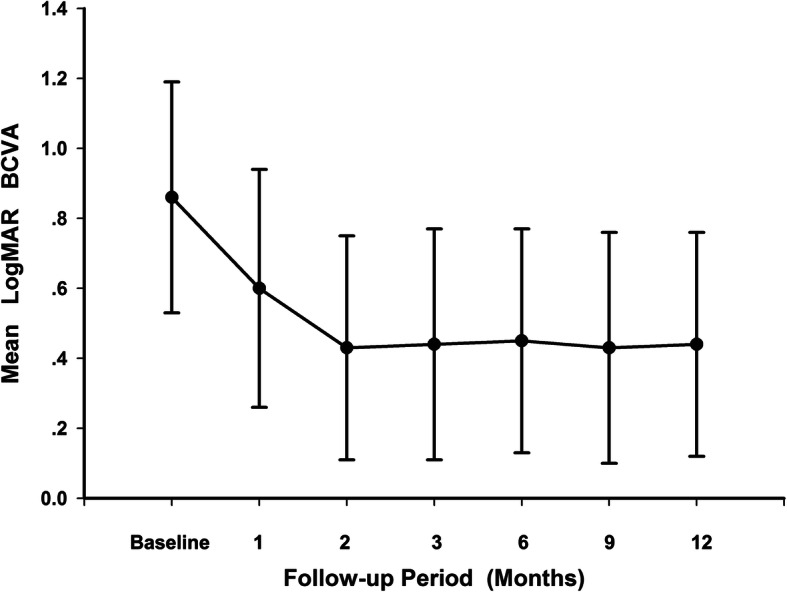
Mean change in best-corrected visual acuity (BCVA) over 12 months. The significant improvements in vision were observed at each follow-up visit compared with baseline values (*p* < 0.001). The greatest improvement in BCVA was seen within the first 2 months, and the BCVA remained stable afterwards. Error bar represents standard deviation

The mean BCVA logMAR of all patients improved significantly from 0.86 ± 0.33 at baseline to 0.44 ± 0.32 at month 12 (*p* < 0.001). Significant improvements in vision were observed at each follow-up visit compared with baseline values (*p* < 0.001) (Fig. 1). The greatest improvement in BCVA was seen during the first 2 months,and the BCVA remained stable afterwards (Fig. 1). The mean improvement in visual acuity was 4.12 ± 2.69 lines with a range of 0 to 9 lines at month 12. An improvement in BCVA of ≥ 3 lines was seen in nineteen eyes (55.9 %), eight eyes (23.5 %) improved by ≥ 2 but < 3 lines, and three eyes (8.8 %) improved by ≥ 1 but < 2 lines. BCVA was unchanged in four eyes (11.8 %). None of the treated eyes lost ≥ 1 line of vision.

**Fig. 2:**
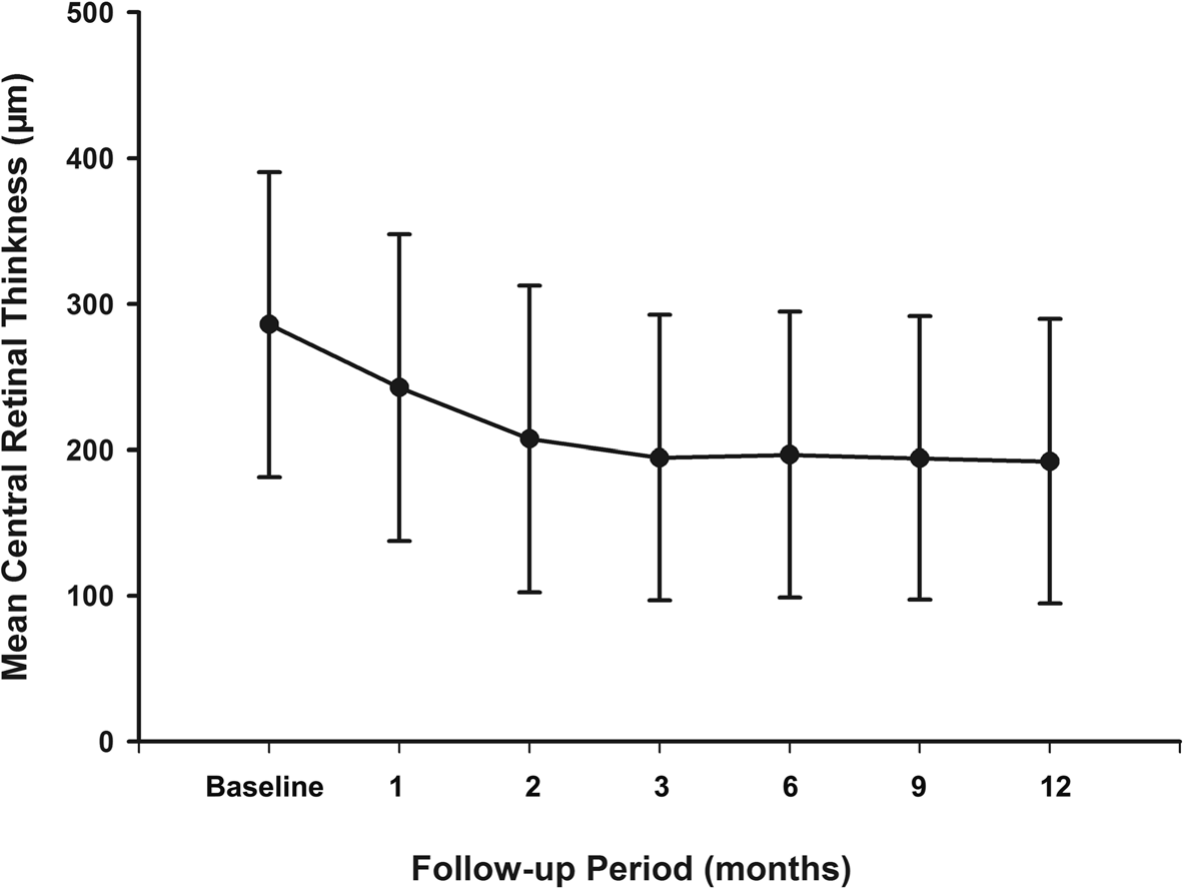
Mean change in central retinal thickness (CRT) over 12 months. The significant improvements in CMT were observed at each follow-up visit compared with baseline values (*p* < 0.001). The greatest improvement in CRT was seen within the first 2 months, and the CRT remained stable afterwards. Error bar represents standard deviation

The mean CRT decreased significantly from 285.9 ± 104.6 µm at baseline to 192.1 ± 97.5 µm at month 12 (*p* < 0.001). Significant improvements in CRT were observed at each follow-up visit compared with baseline values (*p* < 0.001) (Fig. 2). The greatest improvement in CRT was seen during the first 2 months, and the CRT remained stable afterwards (Fig. 2). At month 12, subretinal or intraretinal fluid assessed by OCT disappeared in 32 (94.1 %) eyes.

Finally, in terms of the mean CNV size, patients with intravitreal conbercept showed a reduction from 0.52 ± 0.38 mm^2^ at baseline to 0.31 ± 0.19 mm^2^ at month 12 (*p* < 0.05). FA showed a significant reduction of mean CNV size at month 2 (*p* < 0.05). An absence of CNV angiographic leakage was observed by FA in 32 eyes (94.1 %), while slight leakage persisted in 2 eyes (5.8 %) at month 12. All 34 eyes had reduced or stable size of CNV at the last visit.

### Number of Intravitreal Conbercept injections

Overall, the mean number of IVC was 2.12 ± 0.69 with a range of 1 to 4 in all of the 34 eyes. During the 12-month follow-up, 4 eyes out of the 34 (11.8 %) received one injection, 24 eyes (67.7 %) received two injections, 4 eyes (11.8 %) received three injections, and 2 eyes (5.9 %) received four injections. A total of 28 eyes (82.4 %) needed one or two additional injections in subsequent months after the first injection because of persistent leakage. Two eyes (5.9 %) had recurrence at month 6 and month 9,respectively, and required a total of four injections. These 2 patients had larger subfoveal myopic CNVs.

### Adverse events

There were no death and no case of cerebrovascular event, endophthalmitis, uveitis, or retinal detachment reported in this study. None of the treated patients demonstrated an IOP elevation during any study visit. The most frequent ocular adverse event was conjunctival hemorrhage, which occurred in 4 eyes (4/34, 11.8 %) during the study. Punctate keratitis was reported in 2 eyes (2/34, 5.9 %), and eye pain was reported in 3 eyes (3/34, 8.8 %).

## Discussion

The current study assessed the efficacy and safety of intravitreal conbercept in a 1 + PRN regimen as the primary treatment for myopic CNV. Our results showed that intravitreal conbercept provided significant BCVA improvement with few injections over 12 months. Treatment with conbercept also showed promising anatomical results. CNV size and CRT decreased significantly in 2 months and then remained stable until the end of the follow-up period. No severe systemic or ocular adverse event was observed. Taken together, these outcomes demonstrated efficacy and safety of conbercept treatment for patients with myopic CNV, in a real-world setting.

In recent years, several studies have demonstrated the promising efficacy of anti-VEGF therapies for the treatment of myopic CNV and have established the use of these agents as first-line therapy for this condition. Ranibizumab was the first anti-VEGF drug approved for the treatment of myopic CNV, having been shown to be more effective than PDT. The efficacy of ranibizumab was confirmed by the RADIANCE study, which demonstrated that BCVA gains were significantly superior with intravitreal ranibizumab than PDT, up to week 12, and that ranibizumab treatment alone was effective in further improving and sustaining BCVA in pathologic myopic patients with CNV over 48 weeks [5]. In the REPAIR study, patients received a single injection of ranibizumab followed by a PRN strategy. There was a mean BCVA improvement of 13.8 letters after 48 weeks with a median of 3 injections [6]. Aflibercept has also been reported to be useful for the treatment of CNV secondary to PM. The MYRROR study reported a mean BCVA increase of 13.5 letters in aflibercept-treated patients compared with + 3.9 letters in sham-treated patients. Patients received a median of 2.6 injections within the first 12 weeks and visual improvement was maintained for up to 48 weeks [8].

Conbercept is a novel anti-VEGF agent with several structural similarities to aflibercept. Conbercept binds VEGF-C, in addition to VEGF-A, VEGF-B, and placenta growth factor (PIGF). Conbercept and aflibercept differ structurally in that conbercept contains a fourth VEGFR-2 binding domain, which is thought to enhance the association rate of VEGF to the receptor and prolong its half-life in the vitreous [12, 13]. The results of our retrospective study are in general agreement with those noted in the MYRROR trial [8]. In the MYRROR study, patients receiving aflibercept had a mean improvement in BCVA of 2.38 ± 2.61 lines at 12 months, achieved with a mean number of 2.12 ± 0.69 injections. In addition, the BCVA greatest increase was seen during the first 2 months, and the BCVA remained stable afterwards. In our retrospective study of 34 patients receiving conbercept, the mean improvement was 4.12 lines, with 88 % experiencing visual improvement and 79 % of treated eyes demonstrating an improvement of ≥ 2 lines, with a mean of 2.12 injections. All 34 eyes demonstrated improved or stable vision for up to 12 months. Treatment with conbercept also improved anatomical results. The mean CRT and CNV size decreased significantly at the end of follow-up period compared with baseline. All 34 eyes had a reduced or stable size of CNV at the last visit, 32 eyes (94.12 %) showed a complete closure of CNV on FA. In addition, no serious local or systemic adverse event was noted.

Several reports have supported 3 + PRN regimen to treat myopic CNV. However, many other trials have demonstrated that 1 + PRN is also effective. The results of two different initial dosing schedules of intravitreal ranibizumab for the treatment of CNV secondary to PM have been compared, demonstrating that similar visual improvement was attained in both injection strategies [18]. Moreover, 1 + PRN regimen received 1.37 fewer injections than 3 + PRN regimen over 12 months [11]. These results indicated that one single injection followed by PRN might be a reasonable choice for myopic CNV. Our results also demonstrated that a single initial injection of conbercept for myopic CNV required fewer injections, and provided significant BCVA improvement over 12 months. Four eyes (11.8 %) received one injection, twenty-eight eyes (82.4 %) needed one to two additional injections after the initial injection, and only 2 eyes (5.9 %) showed recurrence at 6 and 9 months and needed a total of four injections. Most notably, the greatest improvement in BCVA was seen in the first 2 months in our study, with minimal subsequent re-injections. Regarding the lower number of conbercept injections needed, the most likely explanation is the different features of conbercept compared with other anti-VEGF drugs. The lower number of injections needed could also be explained by the reduced aggressiveness of CNV secondary to PM, compared with other causes of CNV. Indeed, myopic CNV is different from other indications for anti-VEGF therapeutics, such as nAMD or DME, for which a proactive treatment is required to achieve sustainable and optimal efficacy. However, even in the case of CNV due to nAMD, the AURORA trial demonstrated that conbercept sustained visual improvement with fewr injections, within a 12-month follow-up period [16].

Regarding the prognostic factors, several studies pointed out that the total number of injections was significantly associated with age, myopic refraction, CNV size, and CNV location at baseline [19]. The total number of injections may be another indicator of myopic CNV activity as well as its recurrence. In the current study, focusing mostly on subfoveal CNV (88.23 % of patients), only four eyes (11.8 %) showed a complete resolution of CNV activity after just one IVC. This resolution rate is markedly lower than the resolution rate of 54.7 % reported by Bruè C et al., [20] who focused on both subfoveal and extrafoveal myopic CNV. In addition, in our study, 2 eyes (5.9 %) showed recurrence and needed four injections. These two patients had larger subfoveal CNVs. Thus, our study indicated that subfoveal CNV lesion and larger CNV lesion tended to need a higher number of injections.

The limitations of the current study included its retrospective nature and the relatively small sample size. It also lacked a control group, which is acceptable for a pilot study. Despite these limitations, our results are very promising and support the need for further investigations.

## Conclusions

This retrospective study showed that 1 + PRN intravitreal conbercept is a safe and effective treatment for myopic CNV, and that the resulting visual improvement can be maintained over 12 months. However, our study was retrospective, had a small sample size, and was conducted in a single ophthalmologic institution. Further multi-center, randomized, long-term, and controlled studies are needed to validate these findings.

## Data Availability

The datasets used and/or analyzed during the current study available from the corresponding author on reasonable request.

## Abbreviations

BCVA: Best-corrected visual acuity
CNV: Choroidal neovascularization
CRT: Central retinal thickness
FA: Fluorescein angiography
LogMAR: Logarithm of the minimum angle of resolution
OCT: Optical coherence tomography
PRN: Pro-re-nata
PM: Pathological myopia
VEGF: Vascular endothelial growth factor
IVC: Intravitreal conbercept
